# Supplementary material for: α-Helix stabilization by co-operative side chain charge-reinforced interactions to phosphoserine in a basic kinase-substrate motif
Source: Biochem J. 2022 Mar 16;479(5):687–700. doi: 10.1042/BCJ20210812 (PMC9022996; doi:10.1042/BCJ20210812)
Supplement: Supplementary Material [file BCJ-479-687-s1.pdf]

**α-Helix stabilization by co-operative side chain charge-reinforced interactions to phosphoserine in a basic kinase-substrate motif**

Matthew Batchelor,^+a,b^ Robert S. Dawber,^+a,c^ Andrew J. Wilson,*^a,c^ Richard Bayliss*^a,b^

^a^ Astbury Centre for Structural Molecular Biology, University of Leeds, Woodhouse Lane, Leeds LS2 9JT, UK

^b^ School of Molecular and Cellular Biology, University of Leeds, Woodhouse Lane, Leeds LS2 9JT, UK

^c^ School of Chemistry, University of Leeds, Woodhouse Lane, Leeds LS2 9JT, UK

**Supplementary Information**

**
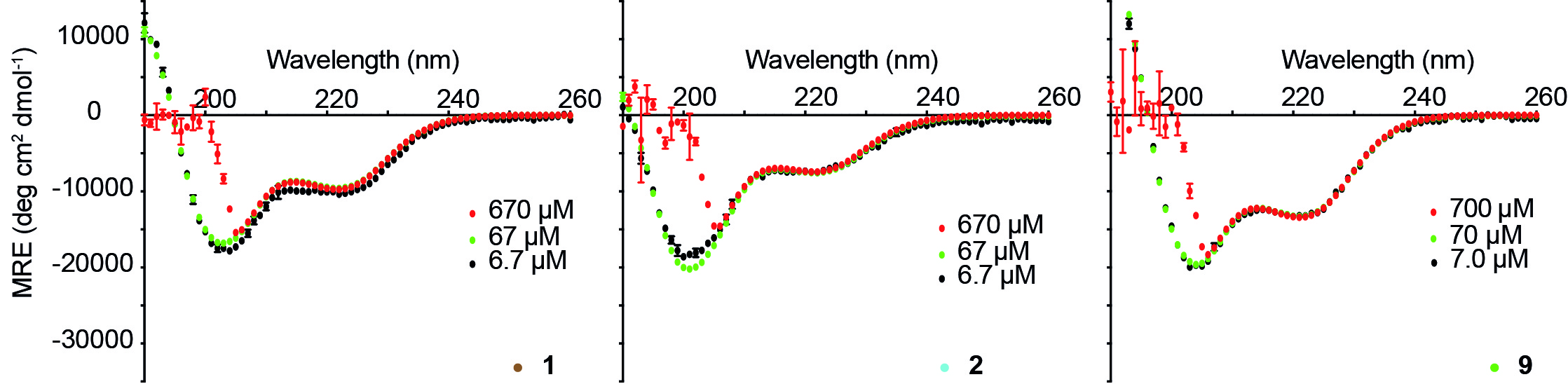
**

**Figure S1.** The effect of concentration on CD spectra for selected peptides (**1, 2** and **9**). Consistent mean residue ellipticity (MRE) profiles over a range of concentrations indicate peptides are monomeric. At high peptide concentrations, high absorbance leads to inaccuracies in ellipticity measurements below ~207 nm. Experiments were carried out at 5 °C in CD buffer (10 mM NaCl, 1 mM sodium phosphate, 1 mM sodium borate, and 1 mM sodium citrate, pH 7).

**
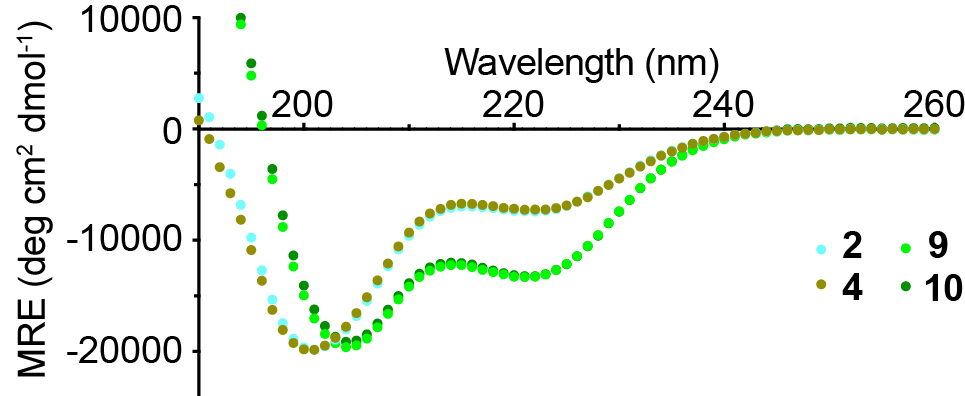
**

**Figure S2.** CD spectroscopy for model peptides **2**, **4**, **9** and **10**. Experiments were carried out at 5 °C in CD buffer (10 mM NaCl, 1 mM sodium phosphate, 1 mM sodium borate, and 1 mM sodium citrate, pH 7). Peptide concentrations were in the range 50–100 μM.

**
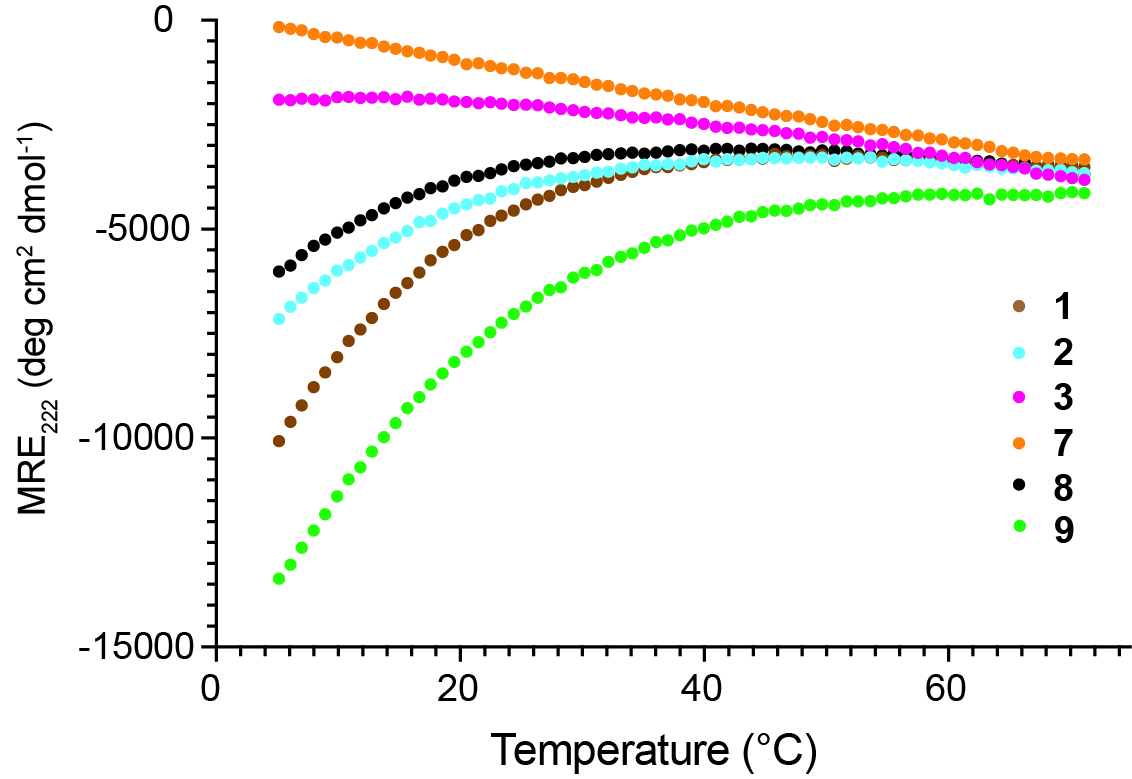
**

**Figure S3.** Temperature dependent CD spectroscopy analyses for selected peptides **1–3** and **7–9** showing the MRE value at 222 nm highlighting a broad transition from part-helical to coil structure. Experiments were carried out in CD buffer (10 mM NaCl, 1 mM sodium phosphate, 1 mM sodium borate, and 1 mM sodium citrate, pH 7). Peptide concentrations were in the range 50–100 μM.

**
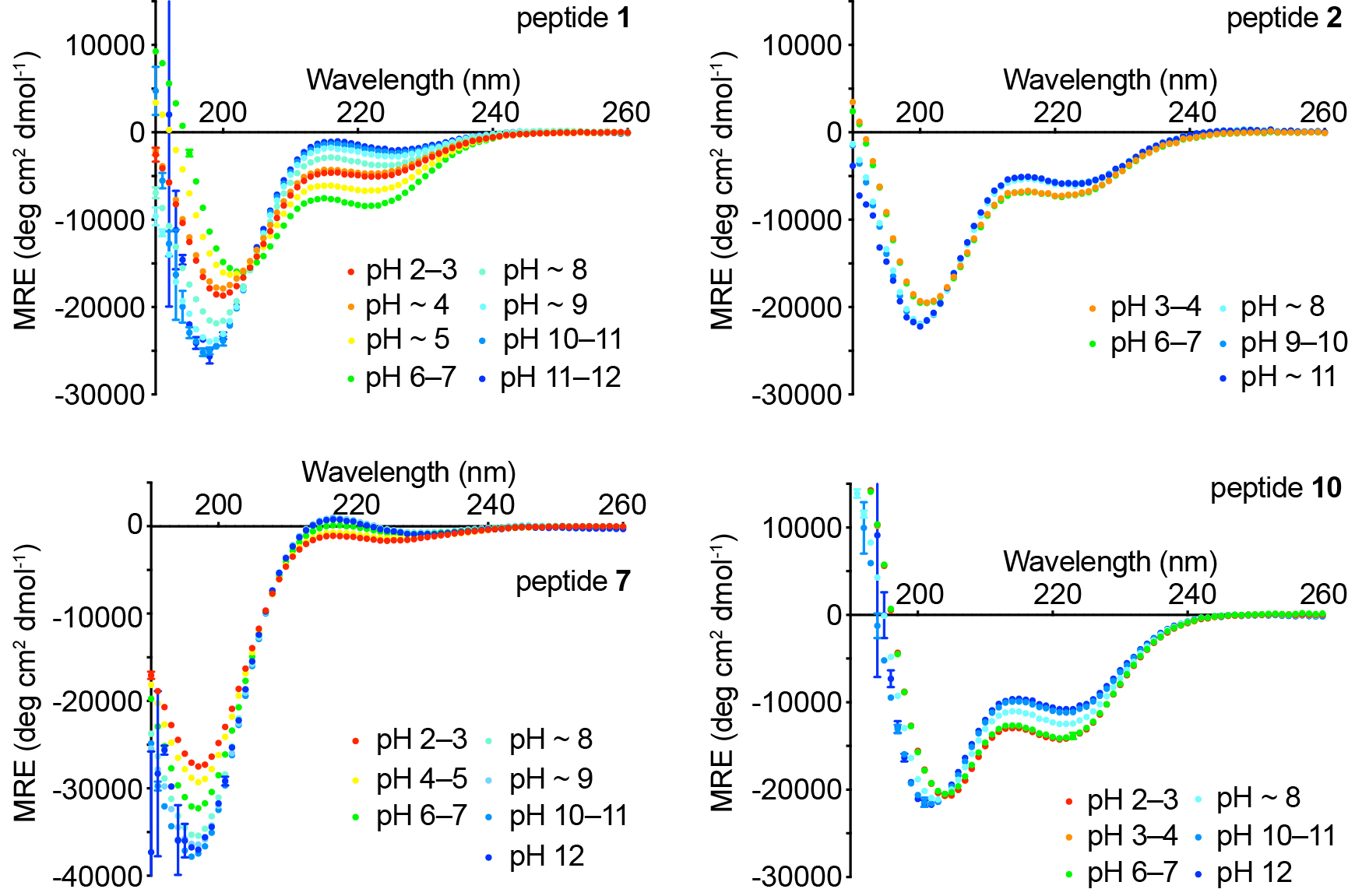
**

**Figure S4.** The effect of pH on a selection of additional peptides (**2**, **7** and **10**). Spectra for peptide **1** are re-shown from Fig. 3 for comparison. Non-phosphate bearing peptides **2** and **10** exhibit a small loss in helicity at high pH and maintain similar structure under low pH and neutral conditions. The pThr containing peptide **7** is ahelical (<2%) across neutral and alkaline pH range and shows a small amount of helicity (~5%) at low pH, suggesting that singly-charged PO_3_H^–^ is slightly less destabilizing than fully deprotonated PO_3_^2–^. Experiments were carried out at 5 °C in CD buffer (10 mM NaCl, 1 mM sodium phosphate, 1 mM sodium borate, and 1 mM sodium citrate). Peptide concentrations were in the range 50–100 μM. The pH was altered by addition of small amounts of 0.1 M HCl or 0.05 M NaOH, with volume changes factored-in to concentration and MRE calculations.


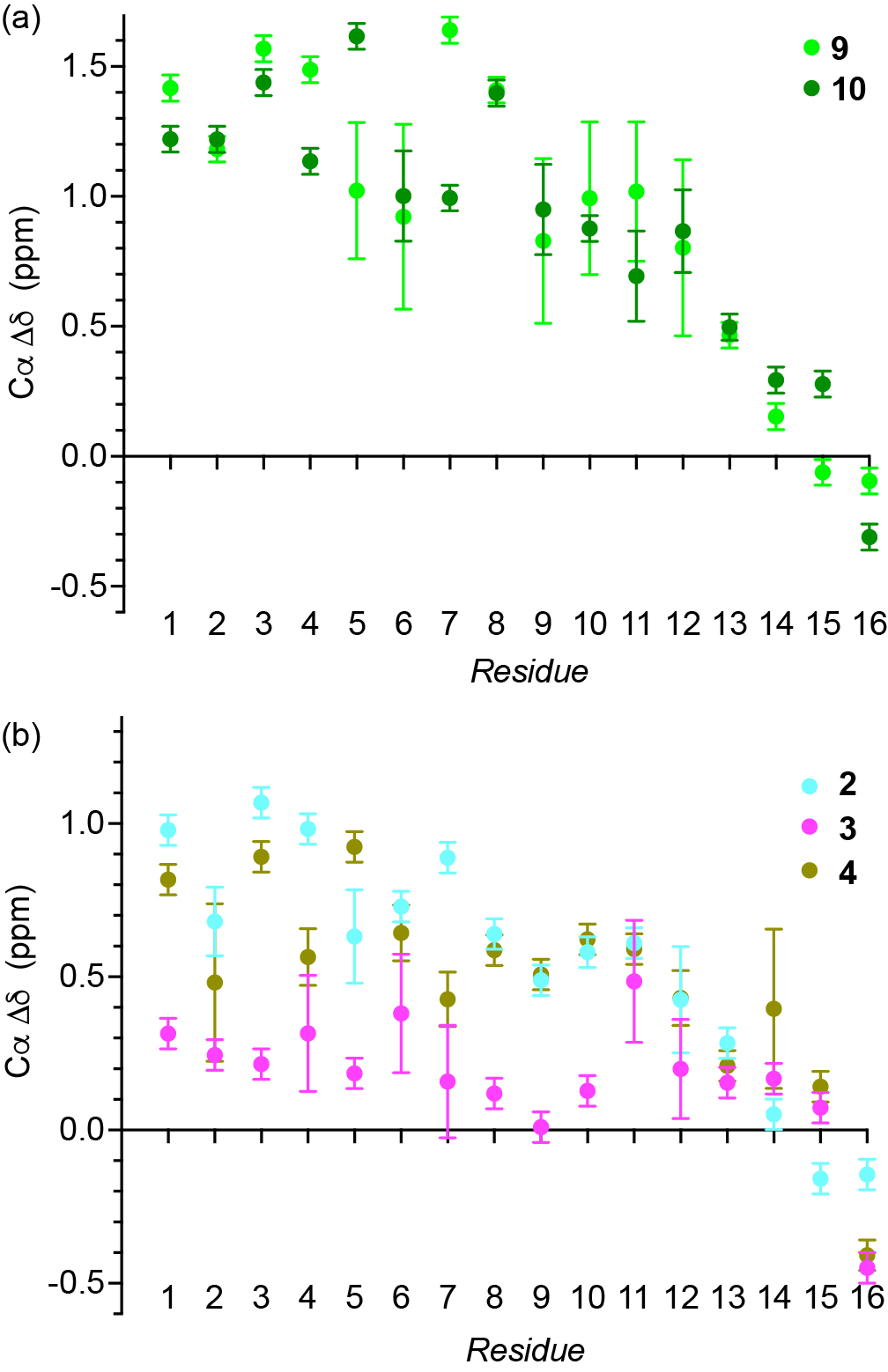


**Figure S5.** Residue specific Cα secondary shifts at 5 °C and pH 6.5–7 are plotted for each residue for (a) Ala control peptides **9** and **10**, and (b) peptide **4**, with secondary shifts for peptides **2** and **3** repeated from Figure. 5 for comparison.


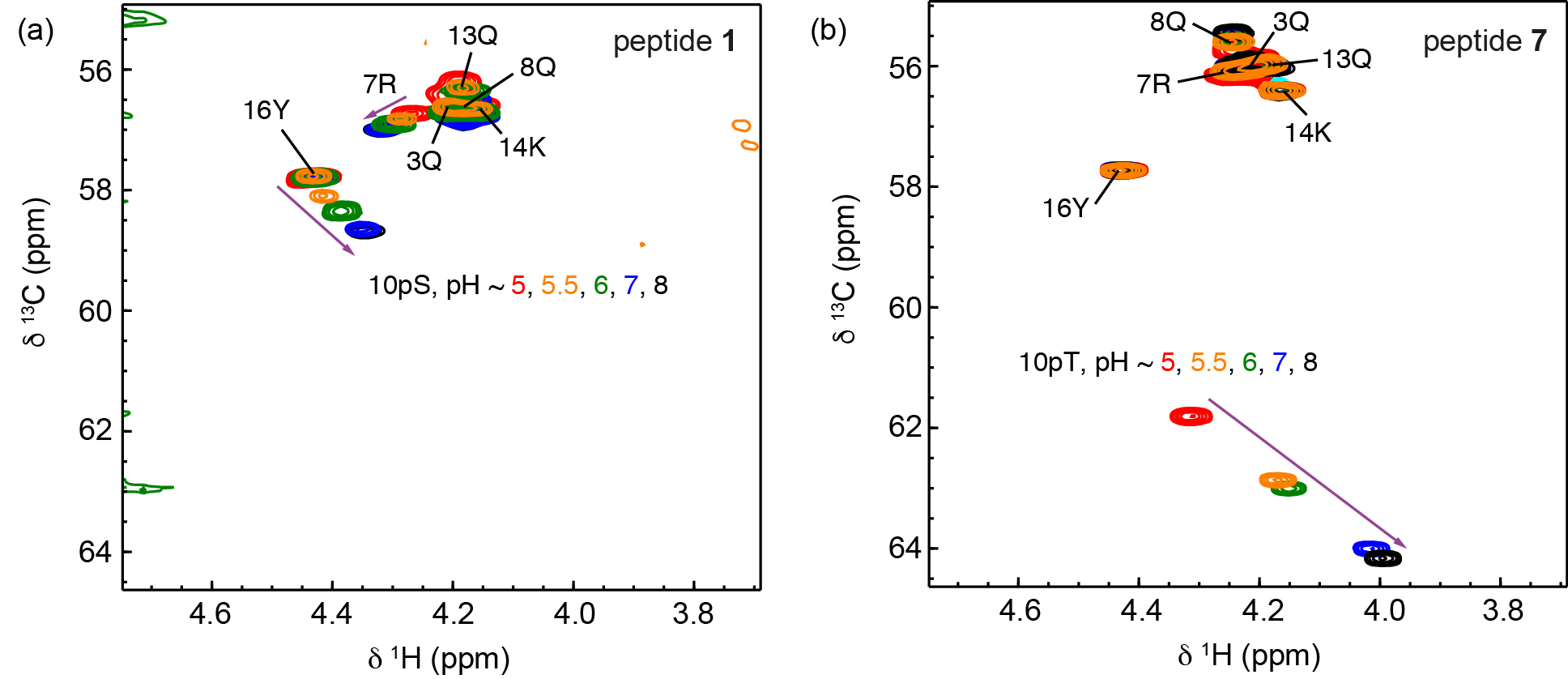


**Figure S6.** Parts of the ^1^H–^13^C HSQC spectra for peptides **1** (a) and **7** (b) at different pH (~5–8). As predicted from published values,^1^ the pSer and pThr Hα–Cα peak changes position with pH. However, the pThr Cα shift values shown in (b) are higher than expected for a residue in a random coil configuration (see the outlier Δδ value for **7** pThr in Fig. 5b), indicating that pThr occupies an arrangement unlike that in the QQpTQQ peptide used to generate the values. With the exception of Arg7 for **1**, which shows a subtle shift, other peak positions shown are largely unchanged with pH, and remain appropriate for an unstructured peptide for **7** and a partially helical peptide in **1**. Spectra were recorded on a 600-MHz Bruker Avance spectrometer equipped with a quadruple resonance QCI-P cryoprobe, or a 950-MHz Bruker Ascend Aeon spectrometer equipped with a 5 mm TXO cryoprobe. Experiments were performed in CD buffer (10 mM NaCl, 1 mM sodium phosphate, 1 mM sodium borate, and 1 mM sodium citrate). The pH was altered by addition of small amounts of 0.1 M HCl or 0.05 M NaOH. Peptide concentrations were in the range 0.5–1.0 mM.

**
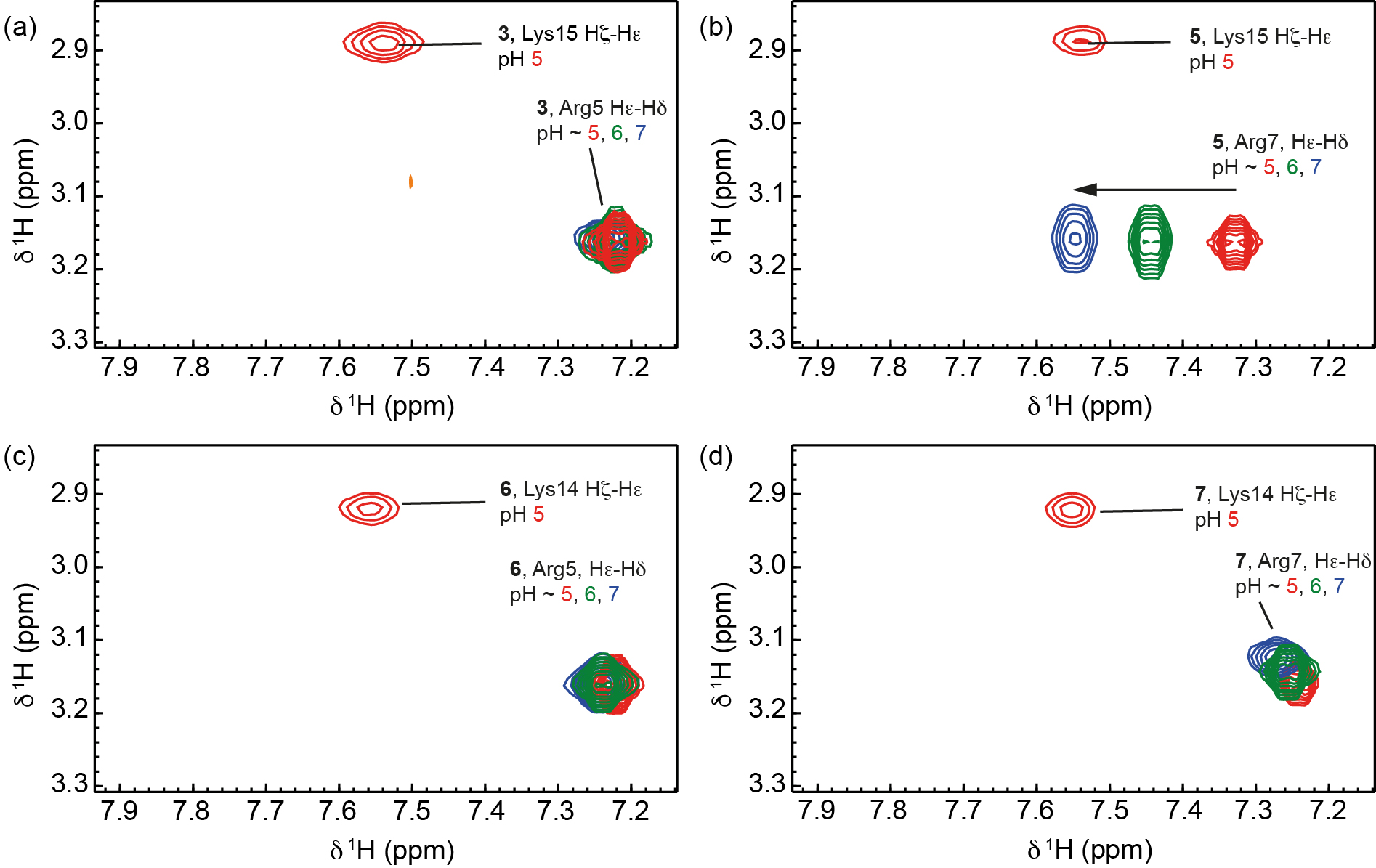
**

**Figure S7.** The region of ^1^H–^1^H TOCSY spectra for peptides **3** (a), **5** (b), **6** (c) and **7** (d) at pH 5–7, showing Arg Hε–Hδ and Lys Hζ–Hε correlations. Only peptide **5** shows an effect of changing pH on the Arg Hε shift, and the Arg Hδ have equivalent resonance positions. Spectra were recorded on a 600-MHz Bruker Avance spectrometer equipped with a quadruple resonance QCI-P cryoprobe. Experiments were performed in CD buffer (10 mM NaCl, 1 mM sodium phosphate, 1 mM sodium borate, and 1 mM sodium citrate). The pH was altered by addition of small amounts of 0.1 M HCl or 0.05 M NaOH. Peptide concentrations were in the range 0.5–1.0 mM.

**
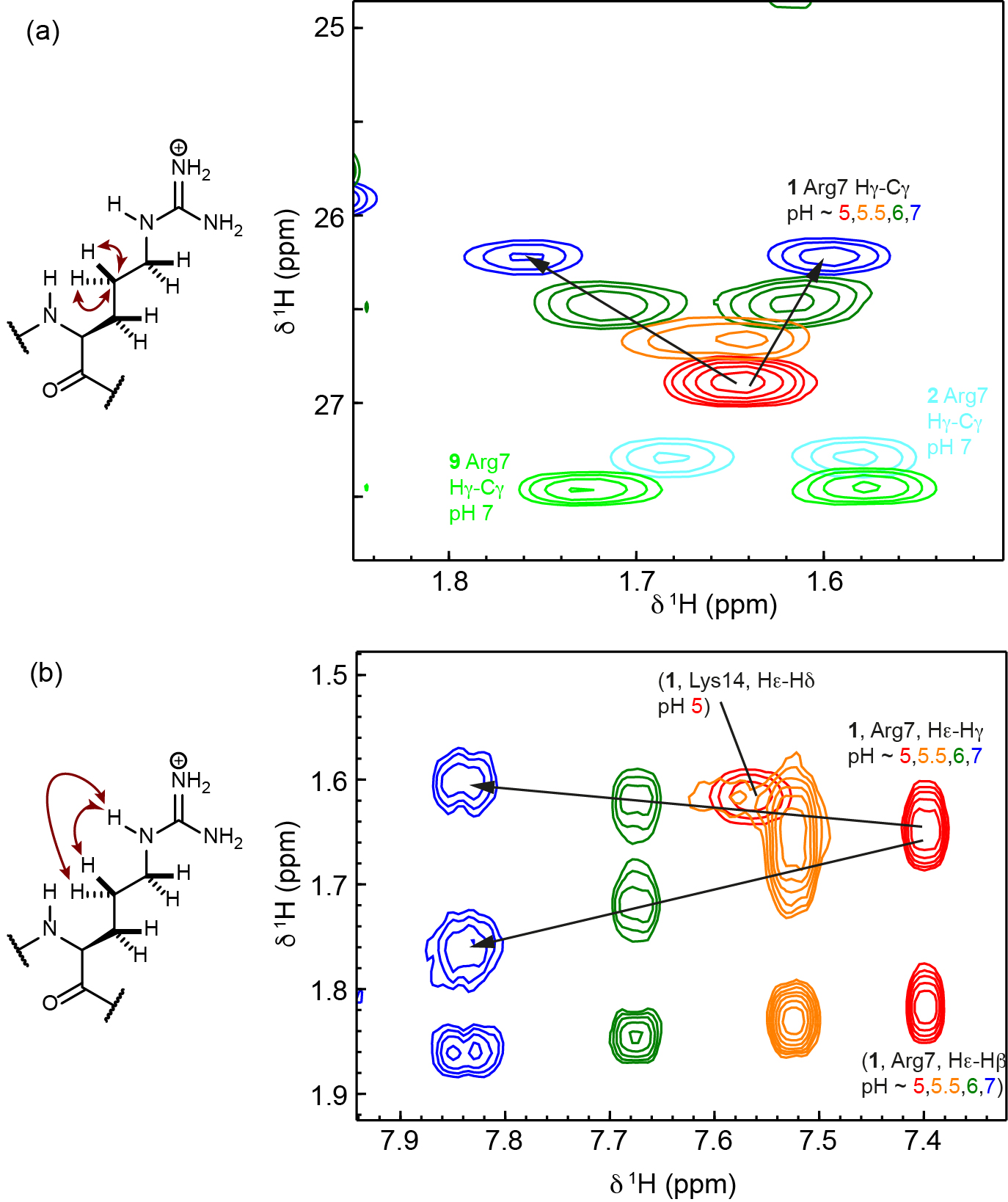
**

**Figure S8.** (a) The region of ^1^H–^13^C HSQC spectra showing Arg Hγ–Cγ correlations for **1** (pH 5–7), **2** and **9** (pH 7). Note the non-equivalence of the two Hγ for peptide **1** at neutral pH as well as for non-phosphorylated but helical peptides **2** and **9**. Low helicity peptides do not show this splitting. (b) The region of ^1^H–^1^H TOCSY spectra for peptide **1** at pH values 5–7, showing Arg Hε–Hγ (and Arg Hε–Hβ) correlations. The Lys Hζ–Hδ correlation is also visible at low pH. Again, the two Hγ for peptide **1** are not equivalent at high pH. Spectra were recorded on a 600-MHz Bruker Avance spectrometer equipped with a quadruple resonance QCI-P cryoprobe. Experiments were performed in CD buffer (10 mM NaCl, 1 mM sodium phosphate, 1 mM sodium borate, and 1 mM sodium citrate). The pH was altered by addition of small amounts of 0.1 M HCl or 0.05 M NaOH. Peptide concentrations were in the range 0.5–1.0 mM.

**Peptide Characterization Data**

**Table S1**. High resolution mass spectrometry data for peptides

| **Peptide** | **[M+H]^1+^ Obs^d^** | **[M+H]^1+^ Exp^d^** | **[M+2H]^2+^ Obs^d^** | **[M+2H]^2+^ Exp^d^** | **[M+3H]^3+^ Obs^d^** | **[M+3H]^3+^ Exp^d^** |
| --- | --- | --- | --- | --- | --- | --- |
| **1** | 1696.8118 | 1696.8046 | 849.4109 | 849.4023 | 566.6102 | 566.6015 |
| **2** | 1616.8452 | 1616.8383 | 809.4296 | 809.4192 | 539.9541 | 539.9461 |
| **3** | 1696.7538 | 1696.8046 | 849.3837 | 849.4023 | 566.5905 | 566.6015 |
| **4** | 1616.8582 | 1616.8383 | 809.4291 | 809.4192 | 539.9527 | 539.9461 |
| **5** | 1696.7538 | 1696.8046 | 849.3841 | 849.4023 | 566.5905 | 566.6015 |
| **6** | 1696.8105 | 1696.8046 | 849.4108 | 849.4023 | 566.6095 | 566.6015 |
| **7** | 1710.8429 | 1710.8203 | 856.4205 | 856.4102 | 571.2816 | 571.2734 |
| **8** | 1630.9757 | 1630.8540 | 816.4368 | 816.4270 | 544.6259 | 544.6180 |
| **9** | 1600.8658 | 1600.8434 | 801.4322 | 801.4217 | 534.6224 | 534.6145 |
| **10** | 1600.8508 | 1600.8434 | 801.4319 | 801.4217 | 534.6231 | 534.6145 |

Peptide **1**


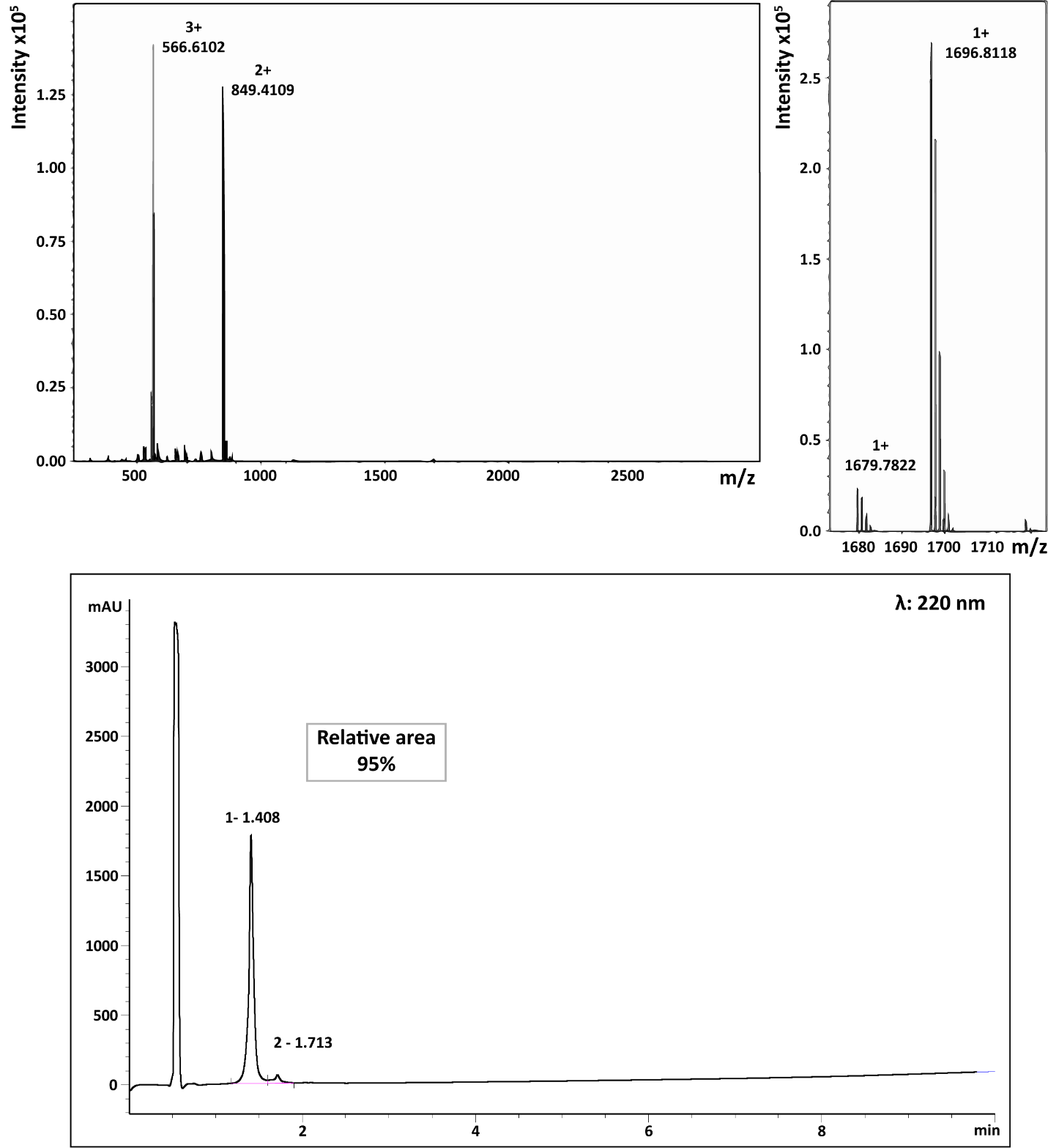


Peptide **2**


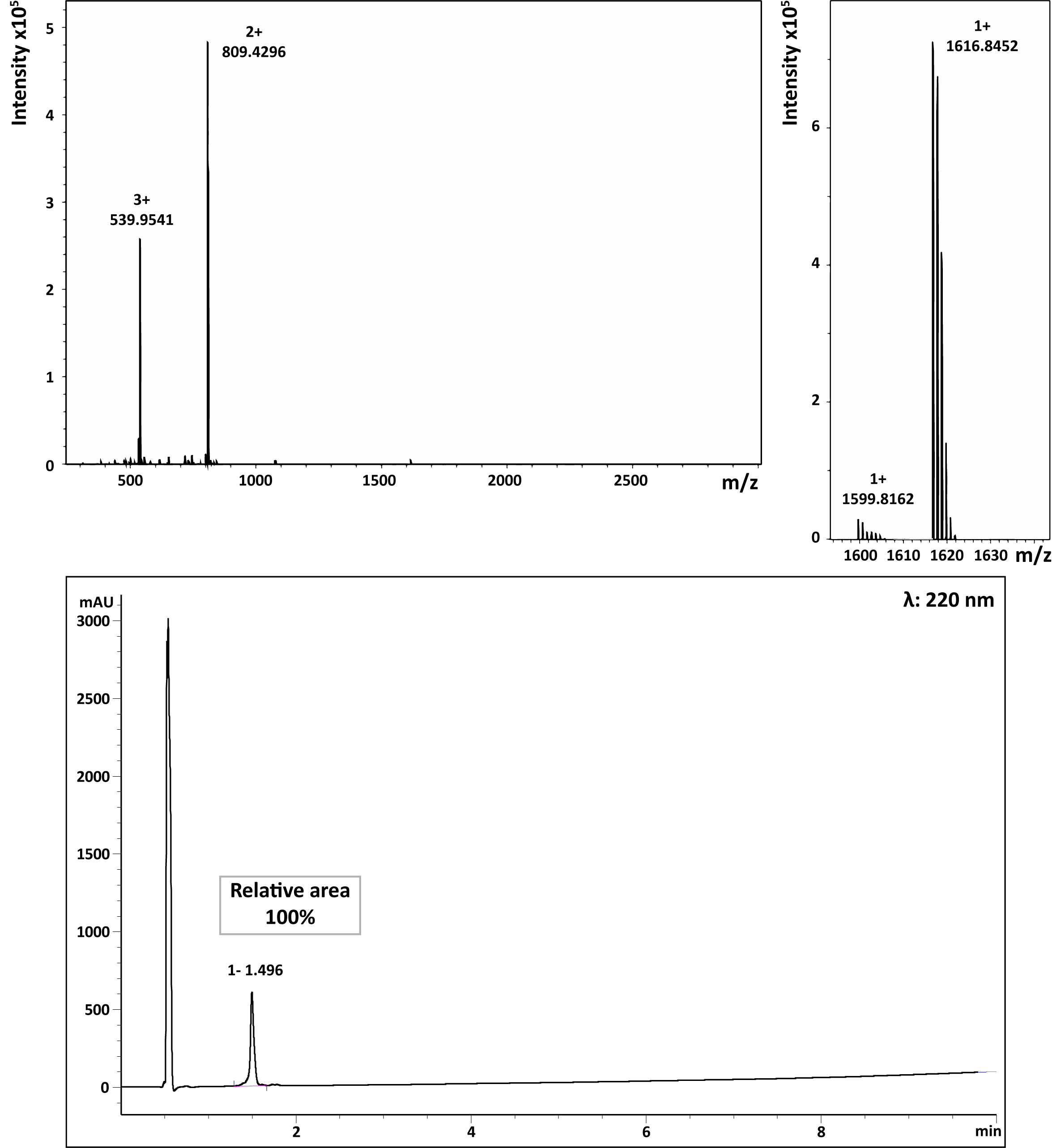


Peptide **3**


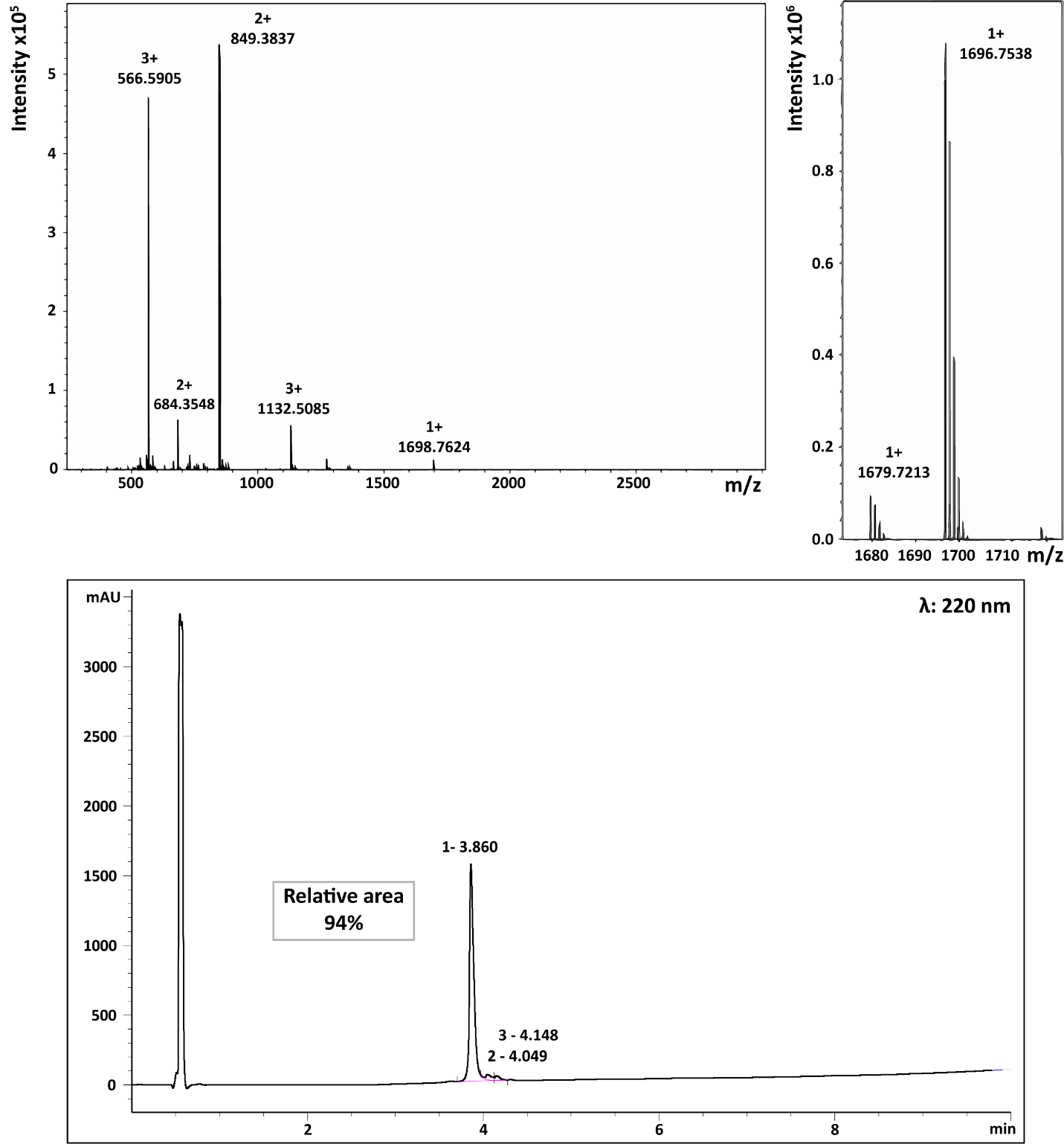


Peptide **4**


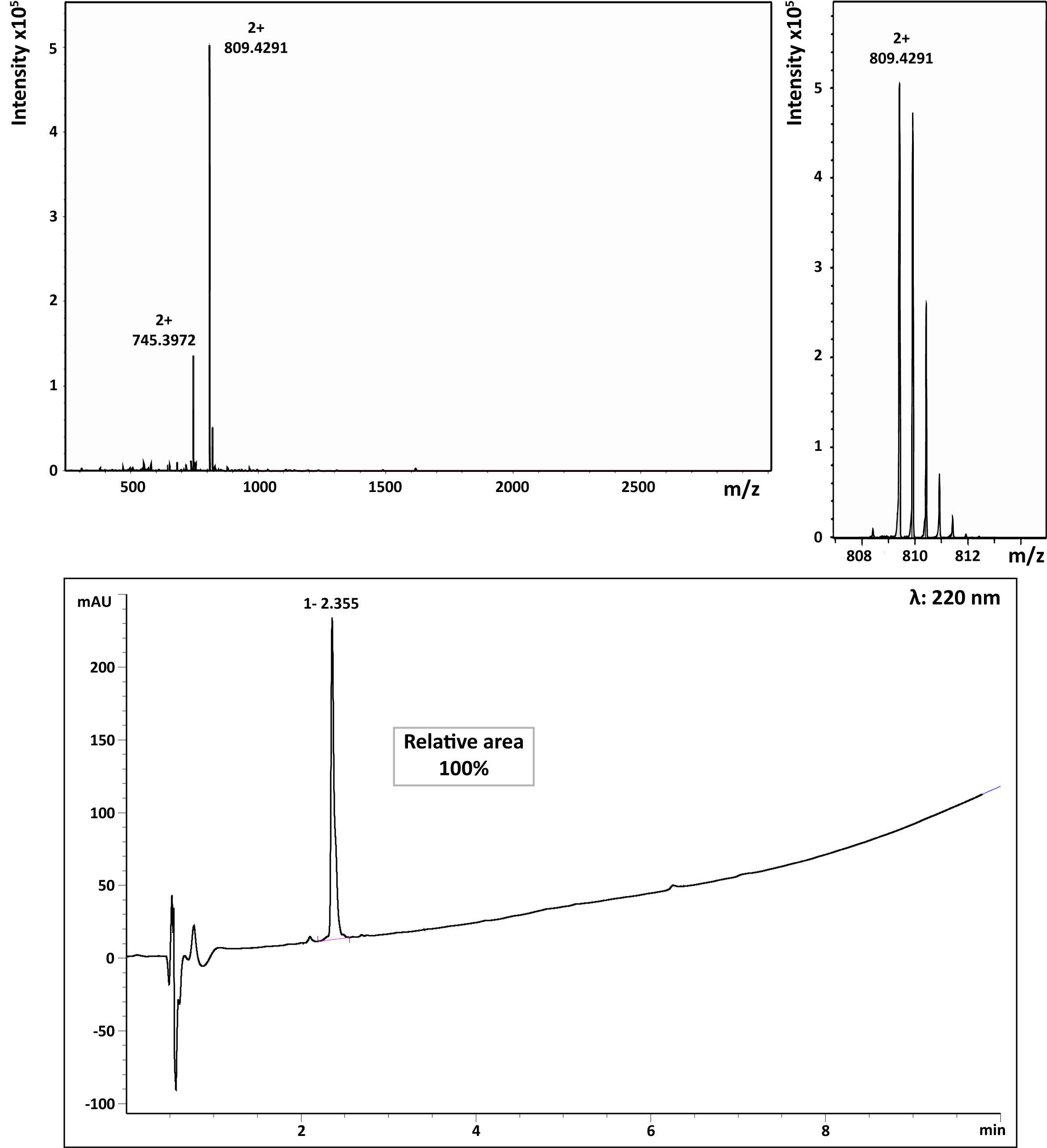


Peptide **5**


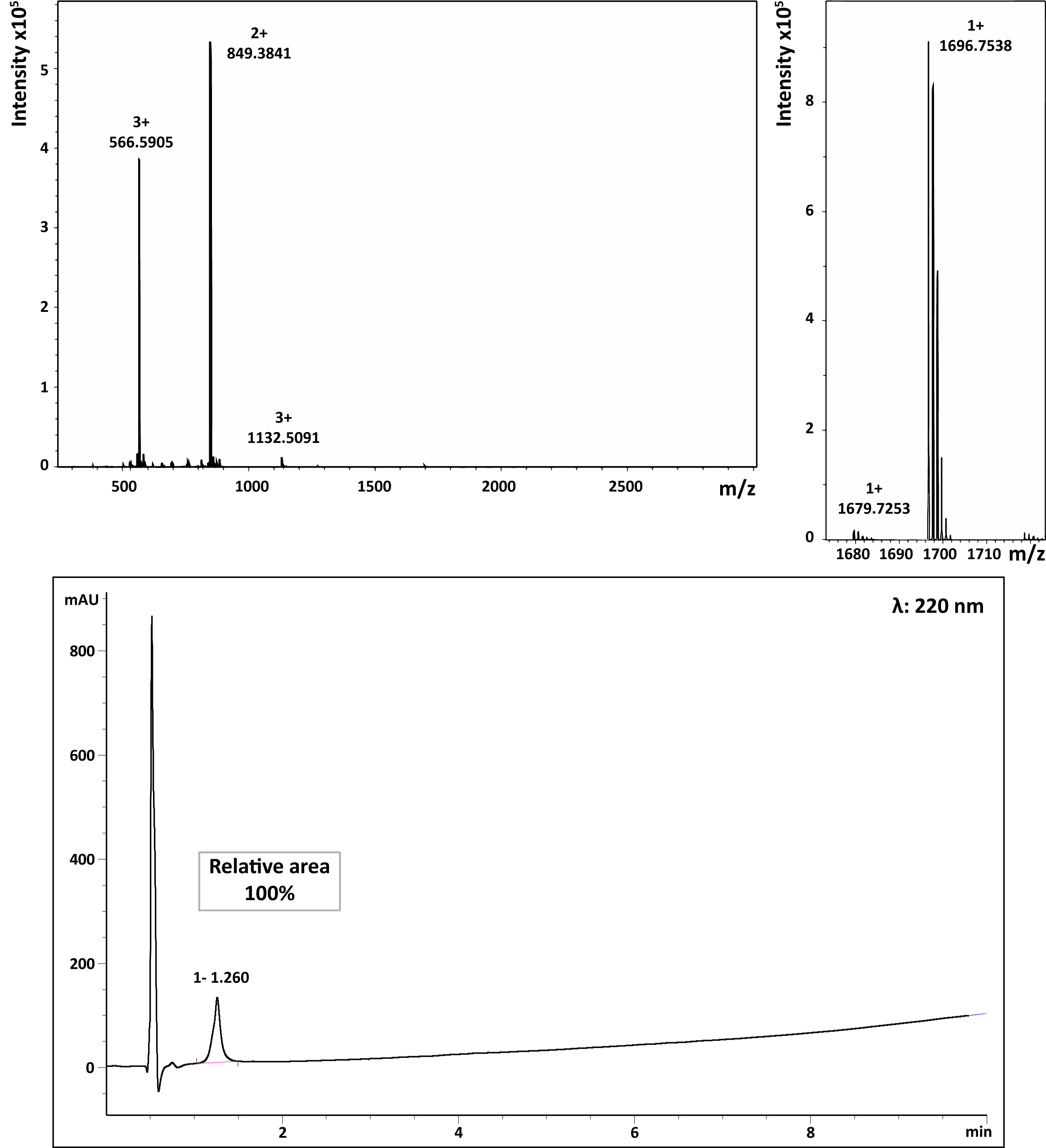


Peptide **6**


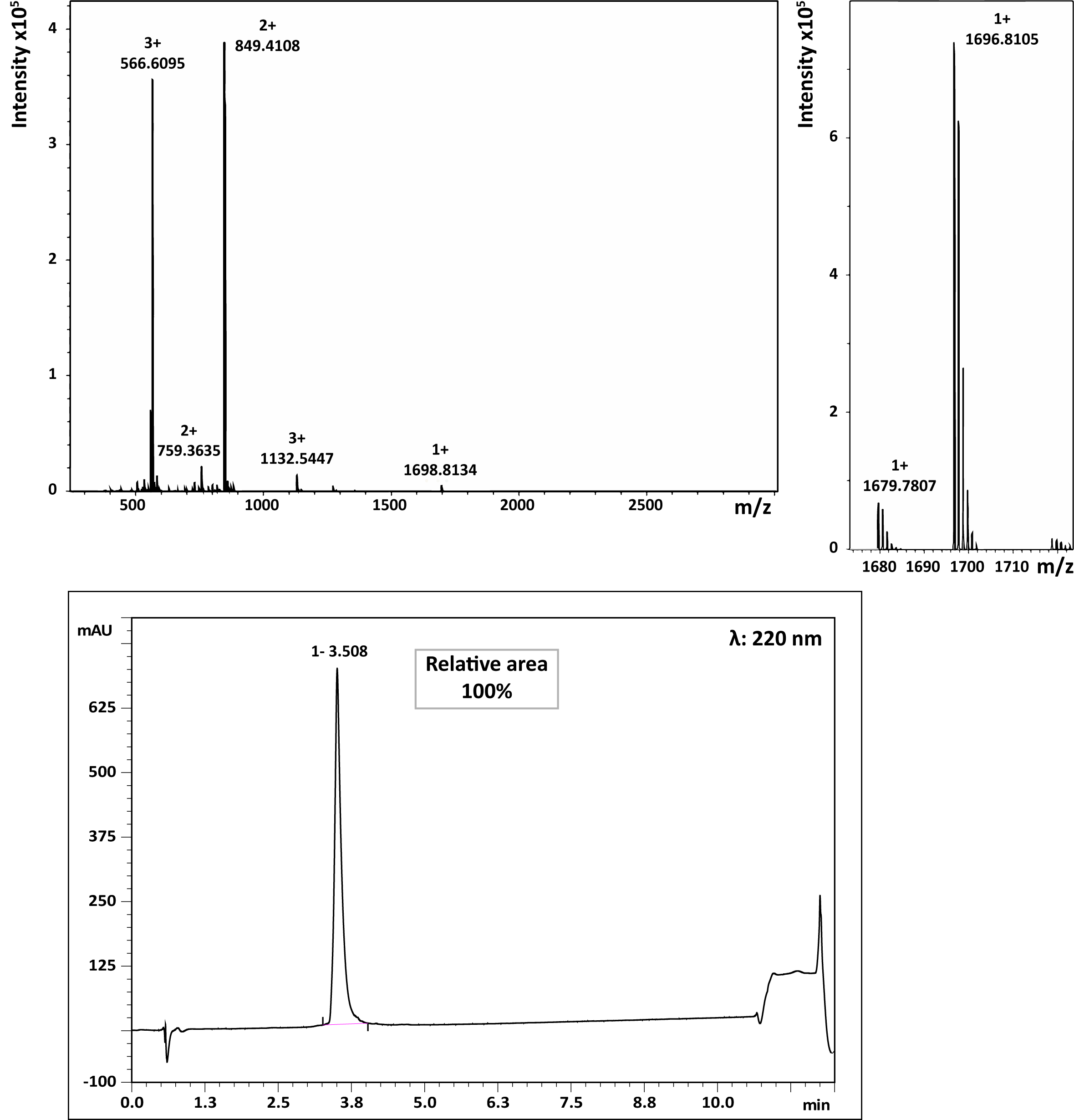


Peptide **7**


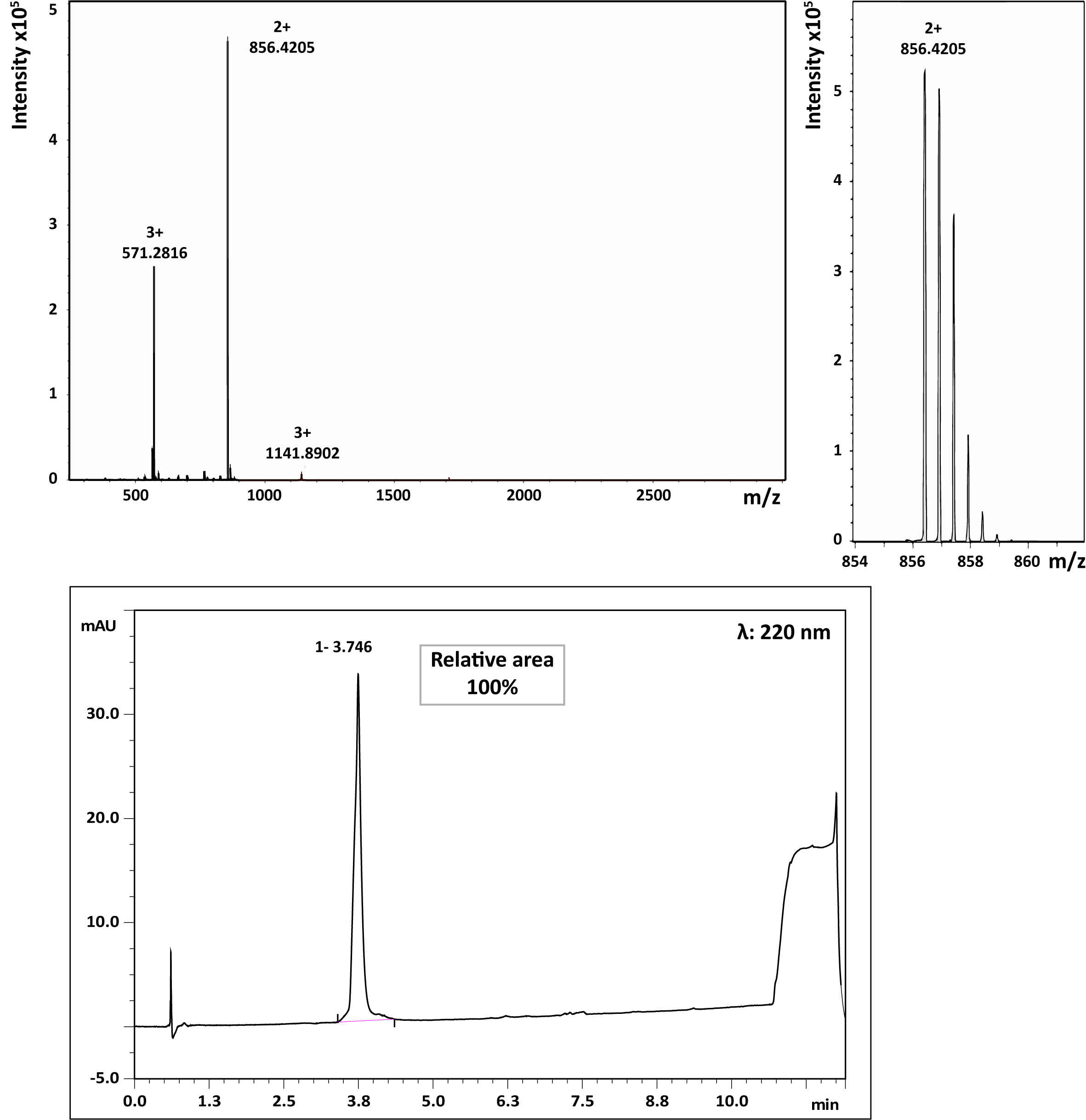


Peptide **8**


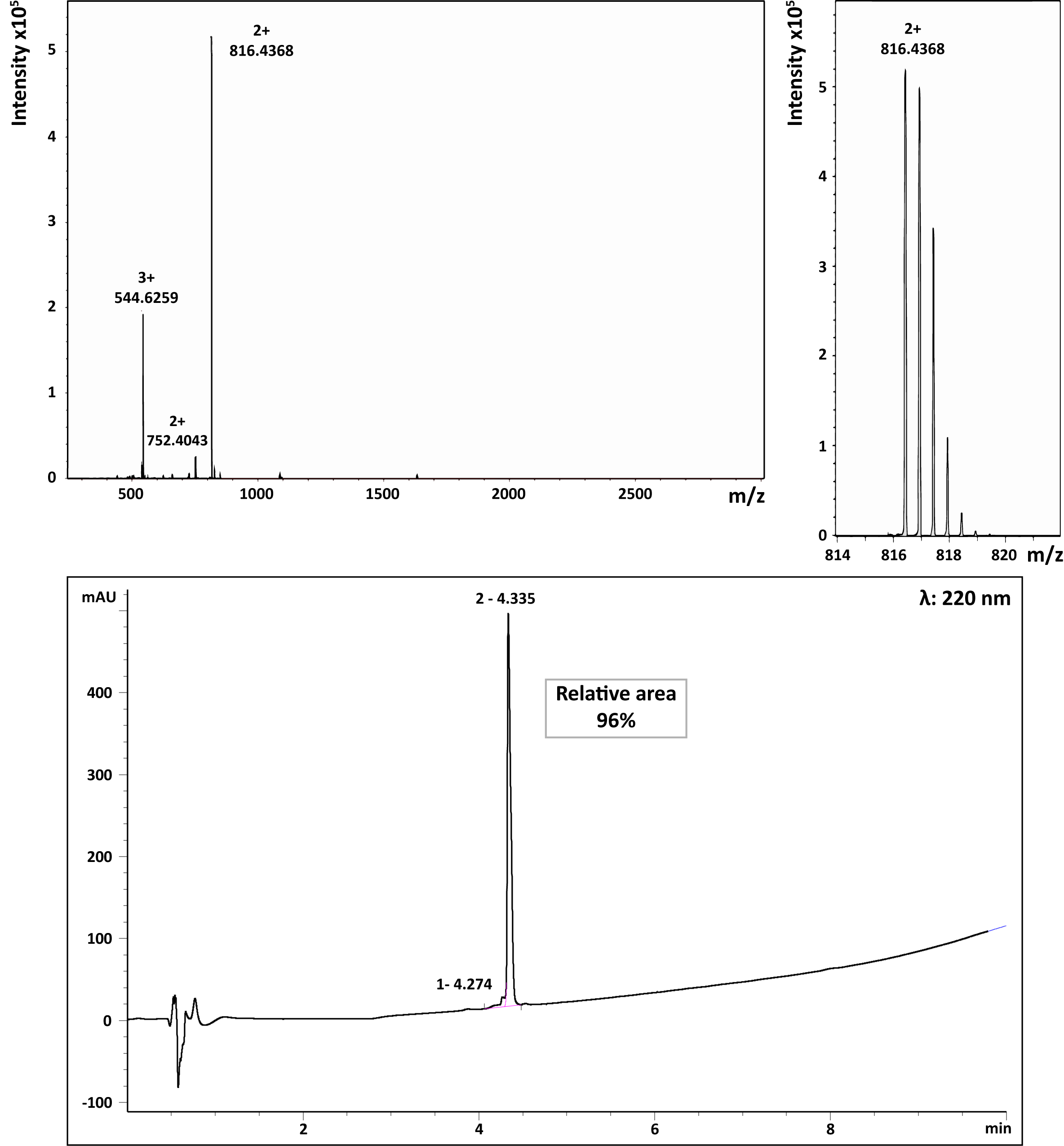


Peptide **9**


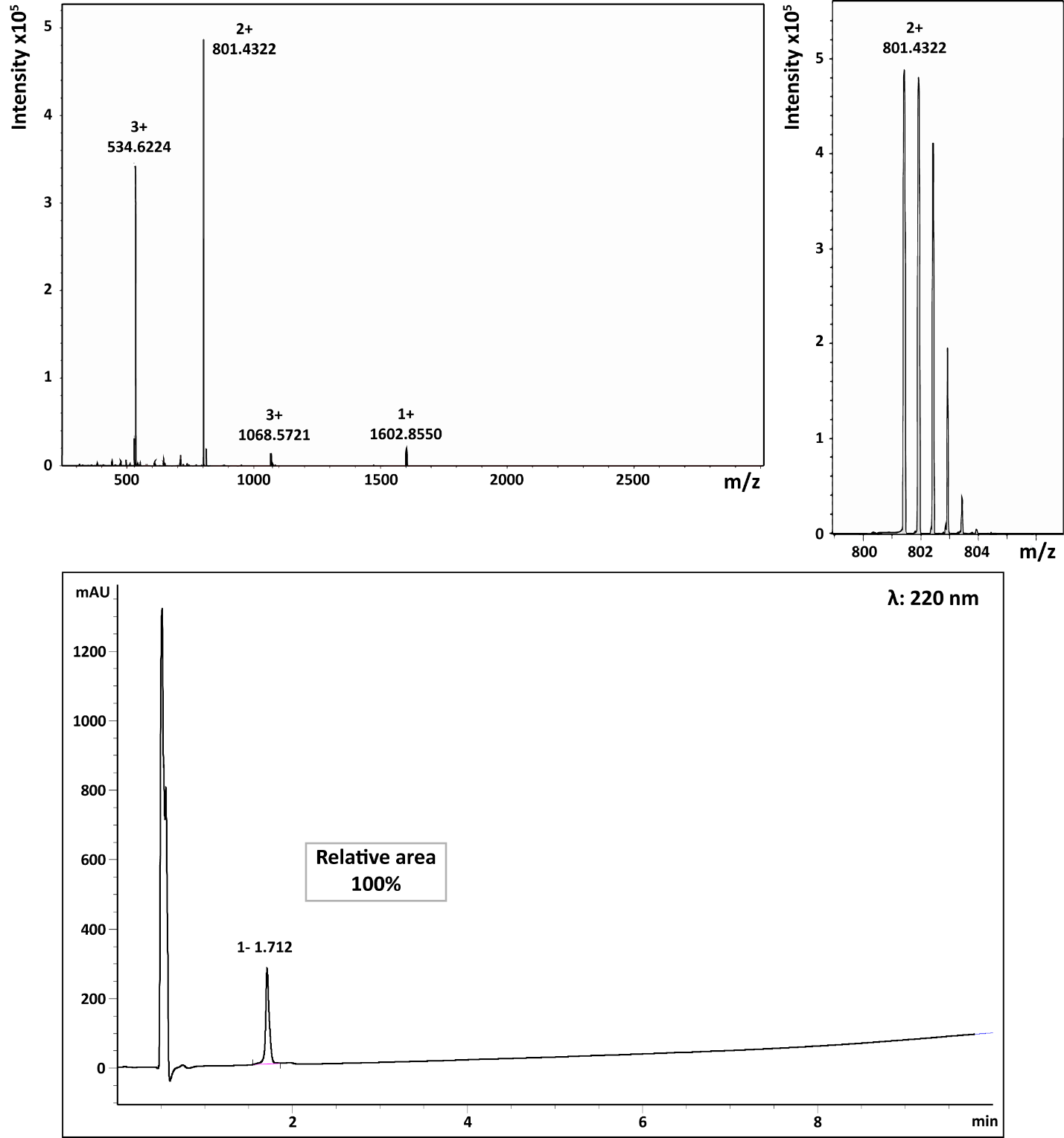


Peptide **10**


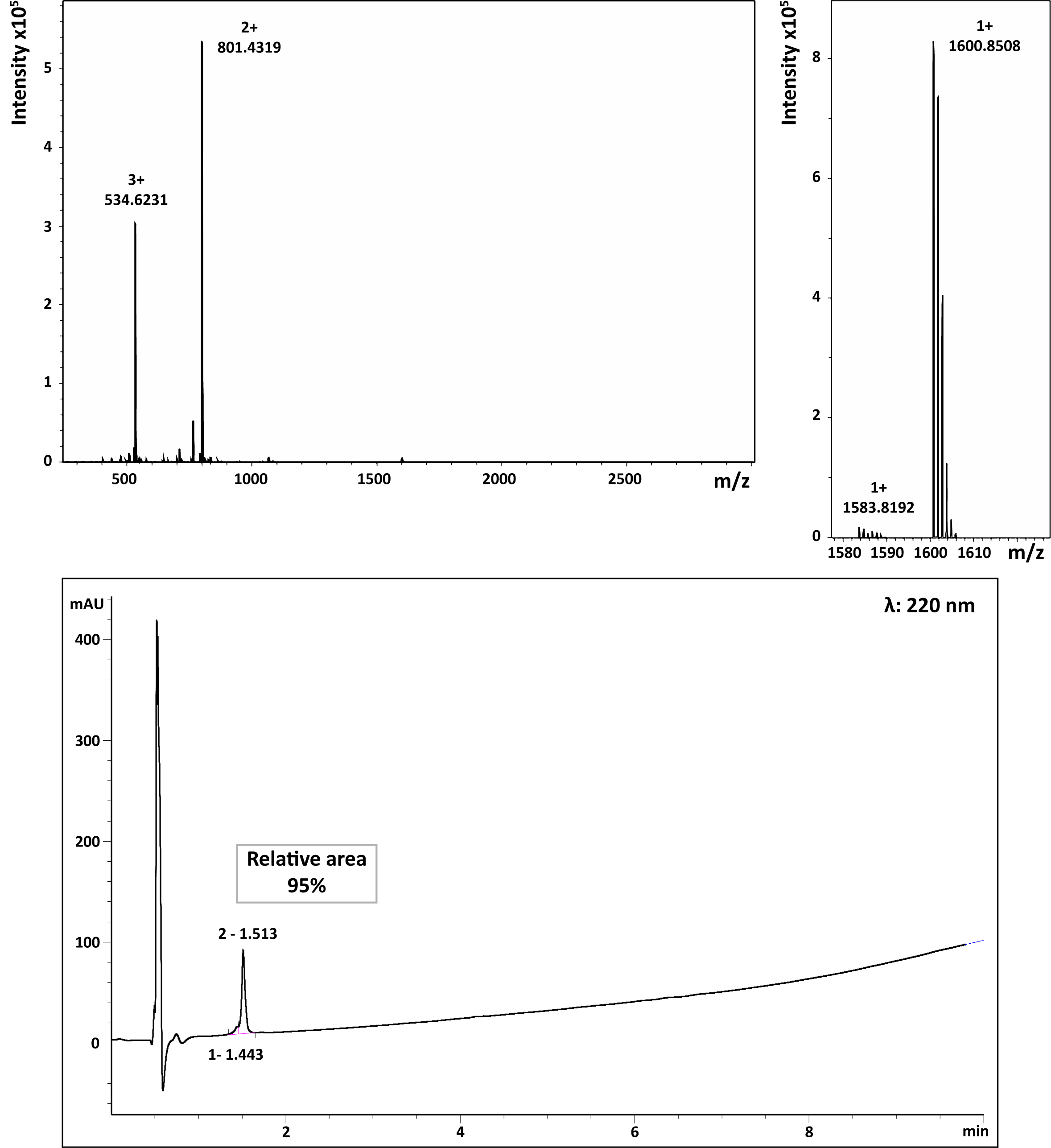


# References

#

1. R. Hendus-Altenburger, C. B. Fernandes, K. Bugge, M. B. A. Kunze, W. Boomsma and B. B. Kragelund, *Journal of Biomolecular NMR*, 2019, **73**, 713-725.
